# The clinical effect and safety of non-pharmacological Chinese medicine therapy in treating chronic nonspecific low back pain: a systematic review and network meta-analysis protocol

**DOI:** 10.3389/fphar.2025.1514231

**Published:** 2025-06-04

**Authors:** Shifang Cui, Lin Liu, Fuli Zhang

**Affiliations:** Heilongjiang University of Chinese Medicine, Harbin, Heilongjiang, China

**Keywords:** non-pharmacological Chinese medicine therapies, chronic nonspecific low back pain, network meta-analysis, protocol, system rview

## Abstract

**Introduction:**

Chronic nonspecific low back pain (CNLBP) significantly affects individual wellbeing, compromising physical health, quality of life, and productivity. As healthcare approaches expand, Non-pharmacological Chinese Medicine Therapies (NPCMT) have garnered global interest. This systematic review and network meta-analysis (NMA) aims to rigorously evaluate the effectiveness and prioritization of various NPCMT interventions for CNLBP, including acupuncture, tui na, gua sha, cupping therapy, taijiquan, baduanjin, yijinjing, and qigong, ultimately providing evidence-based recommendations for healthcare professionals.

**Methods and Analysis:**

Our strategy encompasses a meticulous hunt for randomized controlled trials (RCTs) that evaluate Non-pharmacological Chinese Medicine Therapies for treating chronic nonspecific low back pain. This exploration will span an array of databases, such as Web of Science, Cochrane Library, EMBASE, PubMed, China National Knowledge Infrastructure, China Science and Technology Journal Database, Wanfang Database, China Biology Medicine disc, and ClinicalTrials.gov. Our examination will be comprehensive, encompassing evaluations of risk of bias, meta-analytic procedures, analyses of subgroups and sensitivity, assessments concerning publication bias, and the determination of the quality of evidence.

**Conclusion:**

This study aims to offer valuable insights into the safety and efficacy of various Non-pharmacological Chinese Medicine therapies for patients with CNLBP. It is hoped that these findings will guide future clinical practice and research in identifying the most suitable treatment methods for CNLBP patients.

## Advantages and limitations

1. This will be a network meta-analysis to comprehensively compare different Non-pharmacological Chinese Medicine Therapies for patients with chronic nonspecific low back pain.
2. Published and unpublished studies will be searched to minimise bias.
3. Subgroup analyses, sensitivity analyses, publication bias, and quality of evidence assessments were performed.

## 1 Introduction

Low back pain (LBP), which encompasses a range of symptoms from discomfort and muscle tension to stiffness in the area below the ribs and above the gluteal muscles, occasionally radiating down to the legs, notably as sciatica, is recognized globally as a major health concern (Koes et al., 2006). Research conducted within European demographics indicates that the lifetime prevalence rate for experiencing some form of low back pain can be as high as 84%. Within this context, it is estimated that a subset of individuals, ranging between 5% and 10%, may experience progression of their back pain conditions into chronic low back pain (CLBP) (Denteneer et al., 2015). Furthermore, approximately 90% of these cases are classified as nonspecific low back pain (Dadarkhah et al., 2021). This particular type, chronic nonspecific low back pain (CNLBP), is identified through a clinical presentation of continuous pain or discomfort persisting for 12 weeks or more, in the absence of nerve root disease, specific spinal pathologies, or radicular pain (Ma et al., 2019), including conditions like discogenic pain, lumbar strain, pain in the zygapophyseal joints, and sacroiliac joint pain (Yang et al., 2023). The multifaceted nature of CNLBP, significantly deteriorates the quality of life and work efficiency of affected individuals, culminating in considerable financial repercussions globally (Zhang et al., 2022). Hence, it is critical to allocate increased resources and focus on preventing and treating CNLBP.

Patients diagnosed with this condition are assessed to determine whether they have mild, moderate, or severe pain, based on the intensity of their discomfort. They are then offered pharmacological or non-pharmacological therapies according to their specific level of pain (Ma et al., 2019). Pharmaceutical treatment, a common medical approach, may also be accompanied by persistent issues in treatment efficacy and significant side effects that cannot be overlooked. Common side effects include drowsiness and dizziness, which can adversely impact a patient’s daily life, affecting work efficiency and overall quality of life. More severe side effects, such as organ damage and ulcers, could lead to long-term health complications (Zhang et al., 2024). Research indicates that corticosteroids and antibiotics are useless for CNLBP and pose additional risks to patients. Furthermore, the use of drugs like fluoxetine, topiramate, and gabapentinoids in treating CNLBP shows a high risk of adverse reactions with no significant benefit, coupled with high costs and potential for addiction (Migliorini and Maffulli, 2022). The inconsistent accessibility of traditional treatments has prompted an increasing number of patients to explore alternative methods for symptom relief (Comachio et al., 2020). Non-pharmacological Chinese Medicine Therapy (NPCMT), a vital component of complementary and alternative medicine, has been broadly adopted by traditional chinese medicine (TCM) practitioners for its economic efficiency and effectiveness in addressing chronic nonspecific low back pain, gaining wide patient approval (Yang et al., 2023; Zhang et al., 2024; Comachio et al., 2020; Yeganeh et al., 2017; Golob and Wipf, 2014). NPCMT leverages TCM’s meridian and visceral theories,it aims to holistically modulate the body, heart, and breath, thus enhancing the patient’s mobility, equilibrium, coordination, and overall resilience to the disease as well as their capacity for rehabilitation, marked by few adverse effects, high clinical acceptance, and extensive use in disease prevention and treatment (Fu et al., 2024), including Acupuncture, Tui na, Gua sha, Cupping therapy, Taijiquan, Baduanjin, Yijinjing, and Qigong.

Acupuncture involves inserting thin, sterile needles into specific points on the body. In a study with 76 CNLBP patients, Zhang Afang observed that the acupuncture group notably outperformed the control group. By week 4, acupuncture’s effectiveness reached 100% according to the JOA system, with a significant effect rate of 78.9%. This suggests that acupuncture not only reduces CNLBP pain but also enhances lumbar mobility, showing promising long-term benefits (Zhang, 2020); Tui na uses various manipulative techniques, such as pushing, grasping, kneading, rubbing, and rolling, to stimulate the body’s meridians and acupoints. Han Hui et al. found in their study of 120 CNLBP patients that those receiving Tui na had a significantly higher efficacy rate than the control group, highlighting Tui na’s positive impact on CNLBP (Hui et al., 2005); Gua sha involves the application of repeated pressured strokes on the skin’s surface using a smooth, blunt tool. Liu Zhao et al.'s research involving 150 CNLBP patients indicated that the Gua Sha group experienced significantly lower Visual Analogue Scale (VAS) scores, Oswestry Disability Index (ODI) scores, and clinical symptom scores compared to the control group. VAS is a common method used to assess pain intensity, while ODI is a questionnaire used to evaluate the degree of functional impairment in patients with low back pain. These results prove the effectiveness of Gua Sha in relieving CNLBP pain (Zhao et al., 2015); Cupping therapy typically made of glass, bamboo, or silicone, to create suction on the skin. The cups are placed on the skin, and the air inside is heated and then cooled, creating a vacuum effect. This suction draws the skin upward into the cup, increasing blood flow to the area. Wang Yan et al.'s study on 58 CNLBP patients showed that cupping therapy significantly reduced VAS, ODI, and Roland-Morris Disability Questionnaire (RMQD) Scores, demonstrating its pain-relief effects (Yan et al., 2017); Taijiquan is a traditional Chinese martial art that combines slow, flowing movements, deep breathing, and meditation. The practice involves a series of postures or forms that are performed in a continuous, graceful manner. Liu Jing et al.'s work with 43 CNLBP patients revealed significant improvements in VAS scores following Taijiquan (Liu et al., 2019), which also significantly boosts muscle strength, balance, coordination, proprioception, and mobility of the waist and hip joints, thus enhancing spinal stability (Tao et al., 2023); Baduanjin consists of eight distinct movements or postures, each designed to target specific parts of the body. He Yan et al.'s study involving 120 CNLBP patients found significant reductions in VAS and ODI after 1 and 3 months of practicing Baduanjin, suggesting its efficacy in improving waist pain, function, and flexibility (Yan and Liu, 2020); Yijinjing focuses on stretching and strengthening the muscles and tendons through a series of dynamic and static postures. Liu Mingsheng et al.'s research on 50 CNLBP patients showed that after 12 weeks of Yijinjing, VAS, ODI, abdominal pressure stability, IL-6, TNF-α, 5-HT levels were all lower than those in the control group. This indicates Yijinjing’s potential to enhance both physical and psychological wellbeing in CNLBP patients (Mingsheng et al., 2023); Qigong is a holistic practice that integrates physical postures, breathing techniques, and focused intention. Wang Limin’s study on 64 CNLBP patients demonstrated that, regarding VAS, ODI, and lumbar mobility, the experimental group saw significantly more improvement than the control group. The findings suggest that Qigong can alleviate pain, improve lumbar function and mobility, and enhance the quality of life for CNLBP patients (Wang, 2022).

Systematic reviews and randomized controlled trials lend credence to the effectiveness of these treatments, yet pinpointing the optimal non-pharmacological intervention poses a conundrum in the clinical realm. Network meta-analysis (NMA) is a statistical approach that facilitates the integration of both direct and indirect evidence from various treatment networks, enabling a comprehensive assessment of their relative efficacy. This study aims to scrutinize the efficacy and safety of various NPCMT, including Acupuncture, Tui na, Gua sha, Cupping therapy, Taijiquan, Baduanjin, Yijinjing, and Qigong. By bridging existing research gaps, this investigation seeks to provide clinicians with evidence-based recommendations, aiding in the selection of the most fitting treatment approach.

### 1.1 Objectives

(1) Effectiveness Assessment: Through systematic reviews and network meta-analysis, this study aims to determine the relative effects of various non-pharmacological Chinese medical therapies (NPCMT) in alleviating pain and improving functional impairments in patients with chronic nonspecific low back pain (CNLBP).
(2) Safety Assessment: This analysis will examine the incidence of adverse reactions associated with different NPCMT, providing reference information on safety for clinicians and patients.
(3) Evidence-Based Recommendations: Based on the results related to efficacy and safety, the study will offer evidence-based treatment recommendations for clinical practice, assisting healthcare professionals in selecting the most appropriate NPCMT methods.
(4) Identifying Research Gaps: The study will identify shortcomings and gaps in existing research, guiding future research directions.

## 2 Methods and analysis

Our research approach adheres to the rigorous standards established by the Preferred Reporting Items for Systematic Reviews and Meta-Analyses (PRISMA) and its specific extension tailored for Network Meta-Analyses (Supplementary Material 1) (Moher et al., 2015; Hutton et al., 2015), ensuring our methodology’s transparency, replicability, and comprehensive coverage of the topic at hand. This commitment to high-quality research is further demonstrated by our successful registration with PROSPERO, the international prospective register of systematic reviews, under the registration number CRD42024526076 (NIHR. PROSPERO, 2025).

### 2.1 Search strategy

The search strategy is carefully designed to incorporate both terms from the Medical Subject Headings (MeSH) and free-text searches to guarantee a thorough investigation of pertinent literature, capturing keywords relevant to CNLBP and various treatment methods. The search, grounded in Boolean logic, will cover literature from the databases’ start dates through to May 2025, across a broad spectrum of databases such as Web of Science, Cochrane Library, EMBASE, PubMed, China National Knowledge Infrastructure (CNKI), China Science and Technology Journal Database, Wanfang Database, China Biology Medicine disc, and ClinicalTrials.gov, with no language exclusions. For those interested in the specifics of our approach on PubMed, a detailed description of the search strategy can be found in Supplementary Material 2. We have also extensively reviewed the bibliographies of relevant systematic reviews or meta-analyses to ensure thoroughness.

### 2.2 Eligibility criteria

Utilizing the PICOS framework as a guide, we define our inclusion and exclusion parameters as follows:

Criteria for inclusion: (1) Subjects: Identifying chronic nonspecific low back pain (CNLBP) as lasting more than 12 weeks without radicular lesions, specific spinal pathologies, or radiating pain (diagnostic criteria refer to the definition in Ma et al. (2019), over 18 years old, with no gender or ethnicity restrictions; (2) Interventions: Patients undergoing any non-pharmacological Chinese Medicine therapy, such as Acupuncture, Tui na, Gua sha, Cupping therapy, Taijiquan, Baduanjin, Yijinjing, and Qigong. In this study, we will include research focusing exclusively on single non-pharmacological Chinese medical therapies, excluding studies that employ combined interventions such as acupuncture plus cupping. In cases where a study encompasses both single and combination interventions, data from the sole intervention component will be extracted for analysis; (3) Comparisons: No intervention, placebo, conventional physical therapy, or standard care; (4) Outcome measures: The primary outcome measures include pain and functional impairment. For pain assessment, we will primarily use the Visual Analogue Scale (VAS) as the main analysis indicator, where patients are asked to mark their pain intensity on a straight line, typically 10 cm long, with 0 indicating no pain and 10 representing the most severe pain. This approach enhances the clarity, comparability, and interpretability of the results. For studies that report other pain scales (such as the Numerical Rating Scale (NRS)), we will convert their scores to standardized mean differences (SMD) for uniform comparison with VAS results. The Oswestry Disability Index (ODI) will serve as the primary analysis indicator for functional impairment assessment, whereby patients rate each item based on their actual capabilities, with higher scores indicating greater levels of functional disability. Additionally, results from other functional impairment scales (such as the Roland-Morris Disability Questionnaire (RMDQ)) will also be converted to SMD for analysis. For scales encompassing multiple dimensions, we will select the dimensions most relevant to the core outcomes of this study, based on the research objectives and common practices in the literature. Alternatively, we may summarize scores across various dimensions for inclusion in the analysis. We will carefully document our approach to handling multidimensional scales throughout the data extraction and analysis process to ensure the transparency and reproducibility of the research. Secondary outcomes include the recurrence rate and the incidence of adverse reactions. The recurrence rate refers to the proportion of patients whose symptoms or signs of a disease reappear or worsen within a specified time frame. It reflects both the durability of the treatment effect and the potential risk of disease recurrence. The incidence of adverse reactions indicates the frequency of adverse events related to the intervention that occur during the treatment process. It is essential to monitor and record patients’ subjective reports, clinical observations by healthcare personnel, and laboratory test results. Based on the collected data, the percentage of adverse reaction cases relative to the total number of cases is then calculated; (5) Study Design: randomized controlled trials (RCTs) concentrating on non-pharmacological Chinese Medicine therapies for CNLBP, incorporating all published and unpublished findings of RCTs.

Exclusion criteria: (1) Reviews, systematic reviews, conference abstracts, guidelines, animal research, case reports, duplicates, and unrelated literature; (2) Non-RCTs; (3) Studies that fall outside the parameters of our specified inclusion criteria or fail to provide data that can be reliably extracted will not be considered for inclusion in this analysis.

### 2.3 Study selection

**FIGURE 1:**
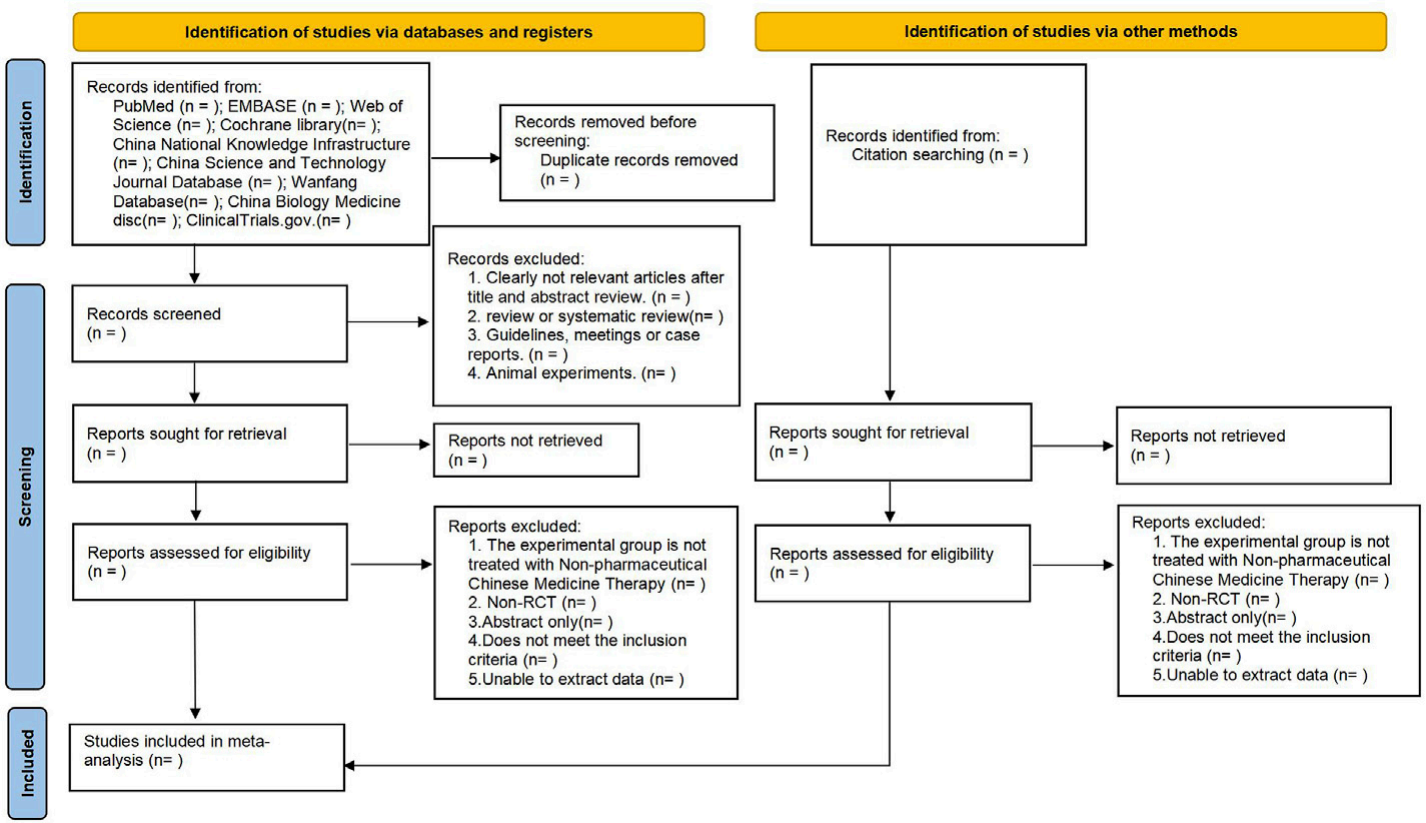
Flowchart.

Search outcomes will be uploaded to the Covidence platform for systematic review processing (Veritas Health Innovation, 2022), where duplicates will be removed. Two reviewers (S.F. Cui, L. Liu) will independently screen titles and abstracts, excluding non-relevant types of studies such as conference abstracts, guidelines, reviews, and unrelated literature. The full texts of the remaining articles will undergo scrutiny against the defined inclusion and exclusion criteria. In instances of disagreement among reviewers, discussions will be held to reach a consensus. Should discrepancies remain unresolved, a third reviewer (F.L. Zhang), will be consulted for a final decision. The selection process will be meticulously recorded, and the PRISMA flowchart will illustrate this process (Figure 1).

### 2.4 Data extraction

In the data extraction phase, authors S.F. Cui and L. Liu will independently gather information using a pre-designed, standardized form, ensuring a systematic approach to data collection. This process will involve a meticulous examination and cross-verification of the extracted data to confirm accuracy and completeness. In cases of disagreement, discussions will be held to reach a consensus, with F.L. Zhang serving as the third reviewer for unresolved disputes. The data to be collected includes study characteristics, participant characteristics, intervention measures, comparator measures, outcome indicators, study quality. Efforts will be made to contact original authors for any missing information to ensure the data’s comprehensiveness.

For continuous outcomes, such as pain levels and functional disability, the average changes from the starting point and their standard deviations. Will be extracted or calculated for both treatment and control groups. For outcomes measured in a binary fashion, such as the frequency of adverse events and rates of recurrence, a comprehensive count of instances and occurrences in both the experimental and control groups will be provided. For studies reporting average pain scores, we will prioritize extracting the means and standard deviations before and after treatment. If only the post-treatment mean or the mean difference is reported, we will attempt to contact the original authors for the missing data. If this information is unavailable, we will use reasonable statistical methods to estimate the values, such as estimating the mean difference based on the correlation coefficient and sample size before and after treatment, to ensure data integrity.

### 2.5 Data items

During the data extraction process, we will collect the following data items from the included studies.

(1) Study Characteristics: Study title, authors, publication year, journal name, type of study design (e.g., randomized controlled trial), inclusion and exclusion criteria for participants, geographical location of the study, and background information.
(2) Participant Characteristics: Sample size, mean age, gender ratio, patients’ baseline characteristics (e.g., pain duration, severity of pain).
(3) Intervention Measures: Specific type(s) of Non-Pharmacological Chinese Medical Therapy (NPCMT), frequency of intervention, duration, course length, methods of implementation, and operational details.
(4) Comparator Measures: Description of the control interventions employed in comparison groups (e.g., no intervention, placebo, conventional physical therapy, standard care).
(5) Outcome Indicators:

Primary outcomes: Pain assessment measures (e.g., Visual Analogue Scale, VAS; Numeric Rating Scale for pain, NRS) and functional impairment assessments (e.g., Oswestry Disability Index, ODI; Roland-Morris Disability Questionnaire, RMDQ).

Secondary outcomes: Recurrence rate, incidence of adverse reactions.

Follow-up time points and frequency.

(6) Study Quality: Methods used for randomization, allocation concealment, blinding procedures applied.

### 2.6 Risk of bias assessment

In the process of assessing the risk of bias, the authors will employ the Cochrane Risk of Bias tool designed for randomized controlled trials (RoB2) (Yang et al., 2017). This tool enables a detailed examination of potential biases within six critical domains. Following this comprehensive evaluation, each study’s risk level will be categorized as “high,” “low,” or “with some concerns,” ensuring a clear understanding of the study’s reliability. In instances where the authors encounter disagreements regarding the bias assessment, discussions will be initiated to resolve these differences. Should these discussions not yield a consensus, the matter will be escalated to F.L. Zhang for final adjudication. The assessment results will be visually summarized in a risk of bias graph.

### 2.7 Statistical analysis

For the statistical analysis component of this research, statistical analyses within this study will be conducted utilizing STATA14.0,to perform meta-analyses. This will involve calculating odds ratios (ORs) for binary outcomes,for continuous variables, we will calculate mean differences (MDs), with the aim of estimating the effect size along with a 95% confidence interval (CI) for each. The determination of statistical significance will play a critical role when P-values are less than 0.05, provided the OR does not encompass one or the MD does not cross 0.

#### 2.7.1 Pairwise meta-analysis

In the specific context of pairwise meta-analyses, data application will follow accordingly from two or more randomized controlled trials that have assessed comparable interventions against similar control conditions. Leveraging the I^2^ statistic to assess study heterogeneity. An I^2^ value under 50% will suggest minimal heterogeneity, warranting a fixed-effect model approach. In contrast, an I^2^ exceeding 50% will indicate substantial heterogeneity, thus requiring a random-effects model (Borenstein et al., 2010). Results will be displayed in forest plots.

#### 2.7.2 Network meta-analysis

Network meta-analyses will further elaborate on these findings by constructing network diagrams that visually compare the efficacy of various interventions. These diagrams will be structured so that the size of each circle represents the sample size of the intervention, and the thickness of the lines between circles reflects the number of studies comparing each intervention. Global consistency and node-splitting methods will be utilized to test for inconsistencies inthe study results, with a P-value of <0.05 indicating inconsistency. Cumulative ranking curve plots, known as SUCRA, will be produced to display the cumulative ranking probabilities of each intervention. Create league tables for indirect two-by-two comparisons of different interventions.

#### 2.7.3 Subgroup and sensitivity analyses

In order to thoroughly investigate and address potential sources of heterogeneity and inconsistency in the Network Meta-analysis (NMA), we will conduct subgroup analyses and sensitivity analyses, which aim to identify factors that may influence the intervention effects and assess the robustness of the study results.

Subgroup analyses will be performed based on several predefined factors, such as intervention duration (short-term vs long-term), patient age (young vs elderly), and intervention types (e.g., different Non-pharmacological Chinese Medicine Therapies). For intervention duration, we will categorize studies into short-term interventions (e.g., within 4 weeks) and long-term interventions (e.g., more than 4 weeks) to explore how the length of intervention may affect the treatment outcomes. Regarding patient age, we will divide studies into those involving younger patients (e.g., under 60 years old) and those focusing on elderly patients (e.g., 60 years old and above) to assess whether age-related differences in physiology and pathology may influence the intervention effects. Additionally, we will perform subgroup analyses according to different types of Non-pharmacological Chinese Medicine Therapies to examine their specific efficacy and safety profiles.

Sensitivity analyses will be carried out to evaluate the impact of study quality and other potential sources of heterogeneity on the overall study findings. We will exclude studies one by one or exclude studies with low methodological quality to assess the influence of individual studies on the overall results. If the results show minimal variation after these exclusions, it indicates low sensitivity, suggesting that the study results are reliable. Conversely, significant disparities in the results after exclusions would signify high sensitivity, indicating less reliable findings.

Moreover, to further address heterogeneity and inconsistency in the NMA, we plan to use meta-regression analysis. Meta-regression is a statistical method that allows us to explore the relationship between study-level characteristics (e.g., intervention intensity, patient demographics) and the effect size. By including these characteristics as covariates in the meta-regression model, we can identify potential sources of heterogeneity and quantify their impact on the intervention effects, to gain a deeper understanding of the factors that may explain the observed heterogeneity and inconsistency in the NMA results.

#### 2.7.4 Publication bias

Should the inclusion number reach ten or more studies, funnel plots along with the Egger test will be applied to investigate publication bias (Chaimani et al., 2013; Egger et al., 1997).

#### 2.7.5 Assessment of the quality of evidence

The evidence quality concerning outcomes will be meticulously evaluated using the Confidence in Network Meta-Analysis (CINeMA) tool (Nikolakopoulou et al., 2020). This assessment will span six domains. S.F. Cui and L. Liu will independently evaluate each domain, with any disagreements resolved through discussion or with the intervention of a third reviewer, F.L. Zhang. This approach ensures a thorough evaluation and presentation of the statistical analyses, contributing to the reliability and credibility of the research findings.

#### 2.7.7 Data synthesis

(1) Data Preparation: Extracted data will be compiled into a standardized format to ensure consistency. We will check for any missing data and, if necessary, attempt to contact study authors for clarification or to obtain additional information.
(2) Network Meta-Analysis Model: A network meta-analysis model will be constructed using STATA software. This model will incorporate both direct and indirect evidence from the included studies to estimate the relative effects of different interventions.
(3) Heterogeneity and Inconsistency Assessment: Heterogeneity will be assessed using the I^2^ statistic, while inconsistency will be evaluated through global consistency and node-splitting methods. We will explore potential sources of heterogeneity and inconsistency through subgroup analyses and meta-regression.
(4) Result Integration: Results from different studies will be combined to provide an overall estimate of the effectiveness and safety of each intervention. Rankings of interventions will be generated based on their efficacy and safety profiles, utilizing methods such as Surface Under the Cumulative RANKing (SUCRA).
(5) Presentation of Findings: Results will be displayed in forest plots, league tables, and SUCRA plots to facilitate interpretation.

### 2.8 Amendments

Any modifications to this protocol (e.g., changes to eligibility criteria, outcome measures, or analytic methods) will be.

(1) Documented: Clearly recorded in the PROSPERO registry (CRD42024526076) with justification for the amendment.
(2) Reported: Transparently described in the final manuscript, including the date of modification and its impact on study design.
(3) Peer-reviewed: Major amendments (e.g., adding new interventions) will be discussed with co-authors and peer reviewers prior to implementation.

## 3 Discussion

A network meta-analysis is performed to evaluate different Non-pharmacological Chinese Medicine Therapies in treating CNLBP. Given the scarcity of pertinent reports, this study aims to bridge this gap. The ranking probability of these treatment options is determined based on factors such as pain relief, dysfunction improvement, adverse effect incidence, and recurrence rate. The objective is to establish a foundation for selecting the most optimal therapeutic approach. The primary concerns highlighted in this study revolve around its limitations due to variability in study designs, participant demographics, intervention strategies, and outcome evaluation methods. Such disparities can introduce heterogeneity, complicating the interpretation of results and increasing their uncertainty. Furthermore, the absence of direct comparisons among specific treatments, coupled with weak evidence for certain comparative analyses, could undermine the overall conclusions’ reliability and validity. The differential quality of the included studies further exacerbates these issues, as studies of lower methodological rigor can negatively influence the overall findings’ precision. To mitigate heterogeneity in the study, we plan to conduct subgroup analyses, categorizing participants based on intervention duration (short-term vs long-term) and patient age (younger vs older). These analyses will help us identify factors that may influence variations in intervention effects. Additionally, we will perform sensitivity analyses to assess the robustness of our findings by excluding low-quality studies or the effects of specific populations, thereby ensuring the reliability of our results.

The results of this study will provide clinicians with a hierarchical recommendation of therapies based on efficacy and safety, guiding them in selecting the most suitable non-pharmacological Chinese medical therapies for patients with CNLBP. For example, if the research finds that acupuncture is the most effective intervention for pain relief, while massage excels in improving functional impairments, clinicians can tailor their treatment plans to align with the specific needs and preferences of their patients.

## Data Availability

The original contributions presented in the study are included in the article/Supplementary Material, further inquiries can be directed to the corresponding author.

## References

[B1] Borenstein M. Hedges L. V. Higgins J. P. T. Rothstein H. R. (2010). A basic introduction to fixed-effect and random-effects models for meta-analysis. Res. Synth. Methods 1, 97–111. 10.1002/jrsm.12 26061376

[B2] Chaimani A. Higgins J. P. T. Mavridis D. Spyridonos P. Salanti G. (2013). Graphical tools for network meta-analysis in STATA. PLOS ONE 8, e76654. 10.1371/journal.pone.0076654 24098547 PMC3789683

[B3] Comachio J. Oliveira C. C. Silva I. F. R. Magalhães M. O. Marques A. P. (2020). Effectiveness of manual and electrical acupuncture for chronic non-specific low back pain: a randomized controlled trial. J. Acupunct. Meridian Stud. 13 (3), 87–93. Epub 2020 Mar 26. PMID: 32224119. 10.1016/j.jams.2020.03.064 32224119

[B4] Dadarkhah A. Rezaimoghadam F. Najafi S. Mohebi B. Azarakhsh A. Rezasoltani Z. (2021). Remote versus in-person exercise instruction for chronic nonspecific low back pain lasting 12 Weeks or longer: a randomized clinical trial. J. Natl. Med. Assoc. 113 (3), 278–284. 10.1016/j.jnma.2020.11.016 33349469

[B5] Denteneer L. Stassijns G. De Hertogh W. Truijen S. Jansen N. Van Daele U. (2015). Derivation and validation phase for the development of clinical prediction rules for rehabilitation in chronic nonspecific low back pain patients: study protocol for a randomized controlled trial. Trials 16, 4. 10.1186/1745-6215-16-4 25558975 PMC4326449

[B6] Egger M. Smith G. D. Schneider M. Minder C. (1997). Bias in meta-analysis detected by a simple, graphical test. BMJ 315, 629–634. 10.1136/bmj.315.7109.629 9310563 PMC2127453

[B7] Fu S. Liu F. Zhi X. Wang Y. Liu Y. Chen H. (2024). Applications of functional near-infrared spectroscopy in non-drug therapy of traditional Chinese medicine: a review. Front. Neurosci. 17, 1329738. PMID: 38333602; PMCID: PMC10851877. 10.3389/fnins.2023.1329738 38333602 PMC10851877

[B8] Golob A. L. Wipf J. E. (2014). Low back pain. Med. Clin. North Am. 98 (3), 405–428. 10.1016/j.mcna.2014.01.003 24758954

[B9] Hui H. Li C. Lianlian F. Qin W. (2005). Clinical analysis of 62 cases of chronic nonspecific low back pain treated with massage techniques. Chin. Rehabil. Med. (02), 97–98. 10.13517/j.cnki.ccm.2005.02.012

[B11] Hutton B. Salanti G. Caldwell D. M. Chaimani A. Schmid C. H. Cameron C. (2015). The PRISMA extension statement for reporting of systematic reviews incorporating network meta-analyses of health care interventions: checklist and explanations. Ann. Intern Med. 162, 777–784. 10.7326/M14-2385 26030634

[B12] Koes B. W. van Tulder M. W. Thomas S. (2006). Diagnosis and treatment of low back pain. BMJ 332 (7555), 1430–1434. 10.1136/bmj.332.7555.1430 16777886 PMC1479671

[B13] Liu J. Yeung A. Xiao T. Tian X. Kong Z. Zou L. (2019). Chen-style tai chi for individuals (aged 50 Years old or above) with chronic non-specific low back pain: a randomized controlled trial. Int. J. Environ. Res. Public Health 16 (3), 517. PMID: 30759778; PMCID: PMC6388249. 10.3390/ijerph16030517 30759778 PMC6388249

[B16] Ma K. Zhuang Z. G. Wang L. Liu X. G. Lu L. J. Yang X. Q. (2019). The Chinese association for the study of pain (CASP): consensus on the assessment and management of chronic nonspecific low back pain. Pain Res. Manag. 2019, 8957847. PMID: 31511784; PMCID: PMC6714323. 10.1155/2019/8957847 31511784 PMC6714323

[B17] Migliorini F. Maffulli N. (2022). Choosing the appropriate pharmacotherapy for nonspecific chronic low back pain. J. Orthop. Surg. Res. 17 (1), 556. PMID: 36544200; PMCID: PMC9773490. 10.1186/s13018-022-03426-5 36544200 PMC9773490

[B14] Mingsheng L. Lili Z. Xiaying C. Weihong Z. (2023). The effects of Yijin Jing psychophysical exercises on the physical and psychological functions of patients with chronic nonspecific low back pain. Contemp. Med. China 30 (34), 8–13. 10.3969/j.issn.1674-4721.2023.34.004

[B18] Moher D. Shamseer L. Clarke M. Ghersi D. Liberati A. Petticrew M. (2015). Preferred reporting items for systematic review and meta-analysis protocols (PRISMA-P) 2015 statement. Syst. Rev. 4 (1), 1. 10.1186/2046-4053-4-1 25554246 PMC4320440

[B19] NIHR. PROSPERO (2025). International prospective register of systematic reviews. PROSPERO: International prospective register of systematic reviews.

[B20] Nikolakopoulou A. Higgins J. P. T. Papakonstantinou T. Chaimani A. Del Giovane C. Egger M. (2020). Cinema: an approach for assessing confidence in the results of a network meta-analysis. PLOS Med. 17, e1003082. 10.1371/journal.pmed.1003082 32243458 PMC7122720

[B21] Tao L. Lu Z. Guangyue Y. Bo Y. Ruojing Z. Qisong H. (2023). The effectiveness of Shi's acupuncture method combined with Tai Chi in treating chronic nonspecific low back pain. J. Integr. Traditional Chin. West. Med. 32 (06), 765–769+775. 10.3969/j.issn.1008-8849.2023.06.006

[B22] Veritas Health Innovation (2022). Covidence systematic review software. Available online at: www.covidence.org (Accessed April 20, 2022).

[B23] Wang L. (2022). A clinical study on the Dao Yin technique from “Zhubing Yuanhou Lun” for chronic nonspecific low back pain. Shandong Univ. Traditional Chin. Med. 10.27282/d.cnki.gsdzu.2022.000878

[B24] Yan He Liu M. (2020). Observing the efficacy of standing Ba Duan Jin in treating chronic nonspecific low back pain. J. Hunan Univ. Traditional Chin. Med. 40 (01), 105–108. 10.3969/j.issn.1674-070X.2020.01.023

[B25] Yan W. Bo L. Fei L. Bo C. Zelin C. (2017). “A clinical study on cupping therapy for chronic nonspecific low back pain [C]//Chinese Acupuncture and Moxibustion Society,” in Proceedings of the 2017 world acupuncture and moxibustion conference and the 2017 annual meeting of the Chinese acupuncture and moxibustion society. Health center in beizhakou town, JinNan district (Tianjin City: Tianjin University of Traditional Chinese Medicine; Tianjin Hexi District Rehabilitation Hospital), 2.

[B10] Yang Z. R. Sun F. Zhan S. Y. (2017). [Risk on bias assessment: (2) Revised Cochrane risk of bias tool for individually randomized, parallel group trials (RoB2.0)]. Zhonghua Liu Xing Bing Xue Za Zhi. 38 (9), 1285–1291. 10.3760/cma.j.issn.0254-6450.2017.09.028 28910948

[B26] Yang J. Zhou X. Ma Q. Woods J. T. Mohabbat A. B. Do A. (2023). Efficacy and safety of Tuina for chronic nonspecific low back pain: a PRISMA-compliant systematic review and meta-analysis. Med. Baltim. 102 (9), e33018. PMID: 36862888; PMCID: PMC9981398. 10.1097/MD.0000000000033018 PMC9981398 36862888

[B27] Yeganeh M. Baradaran H. R. Qorbani M. Moradi Y. Dastgiri S. (2017). The effectiveness of acupuncture, acupressure and chiropractic interventions on treatment of chronic nonspecific low back pain in Iran: a systematic review and meta-analysis. Complement. Ther. Clin. Pract. 27, 11–18. Epub 2016 Nov 30. PMID: 28438274. 10.1016/j.ctcp.2016.11.012 28438274

[B28] Zhang A. (2020). The therapeutic value of acupuncture prescriptions in treating chronic nonspecific low back pain. Heilongjiang Tradit. Chin. Med. 49 (01), 310–311. Available Online at: https://kns.cnki.net/kcms2/article/abstract?v=qQX4xeHgc6udvLNQghycZcBLXzOQ3wkjaFw-PYuGN1ye8mbwlmot8CRJuQ3_32bE2rTH4Fv1cxjKgUu0LHLb5N4h3Bd6-_rPrQbR6jft6Byzu_gqianbC0Yl76ZIIn_zE5NaloCaaZWLd1kRNHJwWhZCBpn4odhkG17bxAnT5KwOJo0zUVAtlloD1aopj1YdfgAET8sVeMw=&uniplatform=NZKPT&language=CHS

[B29] Zhang Z. Pasapula M. Wang Z. Edwards K. Norrish A. (2024). The effectiveness of cupping therapy on low back pain: a systematic review and meta-analysis of randomized control trials. Complement. Ther. Med. 80, 103013. Epub 2024 Jan 5. PMID: 38184285. 10.1016/j.ctim.2024.103013 38184285

[B30] Zhang Z. Zhang C. Li Y. Wang C. Yu Q. (2022). Lipid and metabolic alteration involvement in physiotherapy for chronic nonspecific low back pain. Lipids Health Dis. 21 (1), 125. PMID: 36434687; PMCID: PMC9700977. 10.1186/s12944-022-01737-4 36434687 PMC9700977

[B15] Zhao L. Yingying W. Yuan W. Shulan Q. Jinsheng Y. (2015). A randomized controlled trial on scraping therapy for chronic nonspecific low back pain. Chin. J. Traditional Chin. Med. 30 (05), 1458–1463. Available Online at: https://kns.cnki.net/kcms2/article/abstract?v=qQX4xeHgc6uhNXJGOZD7DBD0kOsdpVW6fwukpJ3nvWMEHdDoviGZXRp7gEDCnR0uw8ZrihBMTGV4MFDxRJL4KAvIrOfOAr6qahKSgqO3jcHY-Jctfu5K0dDnjyZOOosGyFBNz0TtpnVLaBVXMQ--Ve9oxGMnl_6cQXuOjlyGxsBz3JwgWEGg3RJRdDbOQwpJxiui72wGXgU=&uniplatform=NZKPT&language=CHS

