# Supplementary material for: The clinical effect and safety of non-pharmacological Chinese medicine therapy in treating chronic nonspecific low back pain: a systematic review and network meta-analysis protocol
Source: Front Pharmacol. 2025 Jun 4;16:1514231. doi: 10.3389/fphar.2025.1514231 (PMC12174129; doi:10.3389/fphar.2025.1514231)
Supplement: Supplementary file 1 [file Table1.docx]

**Supplement 1.Searching strategies**

| **PubMed** | |
| --- | --- |
| #1 | "Low Back Pain"[Mesh] |
| #2 | Back Pain, Low[Title/Abstract] OR Back Pains, Low[Title/Abstract] OR Low Back Pains[Title/Abstract] OR Pain, Low Back[Title/Abstract] OR Pains, Low Back[Title/Abstract] OR Lumbago[Title/Abstract] OR Lower Back Pain[Title/Abstract] OR Back Pain, Lower[Title/Abstract] OR Back Pains, Lower[Title/Abstract] OR Lower Back Pains[Title/Abstract] OR Pain, Lower Back[Title/Abstract] OR Pains, Lower Back[Title/Abstract] OR Low Back Ache[Title/Abstract] OR Ache, Low Back[Title/Abstract] OR Aches, Low Back[Title/Abstract] OR Back Ache, Low[Title/Abstract] OR Back Aches, Low[Title/Abstract] OR Low Back Aches[Title/Abstract] OR Low Backache[Title/Abstract] OR Backache, Low[Title/Abstract] OR Backaches, Low[Title/Abstract] OR Low Backaches[Title/Abstract] OR Low Back Pain, Postural[Title/Abstract] OR Postural Low Back Pain[Title/Abstract] OR Low Back Pain, Posterior Compartment[Title/Abstract] OR Low Back Pain, Recurrent[Title/Abstract] OR Recurrent Low Back Pain[Title/Abstract] OR Low Back Pain, Mechanical[Title/Abstract] OR Mechanical Low Back Pain[Title/Abstract] OR chronic nonspecific low back pain[Title/Abstract] OR chronic nonspecific low back pain[Title/Abstract] OR chronic nonspecific lumbago[Title/Abstract] OR chronic nonspecific lower back pain[Title/Abstract] OR chronic nonspecific lumbar pain[Title/Abstract] |
| #3 | #1 OR #2 |
| #4 | acumoxi[Title/Abstract] OR acupuncture and moxibustion[Title/Abstract] OR acupuncture[Title/Abstract] OR manipulation[Title/Abstract] OR massage[Title/Abstract] OR scraping[Title/Abstract] OR ventouse[Title/Abstract] OR cupping glass[Title/Abstract] OR taich[Title/Abstract] OR taijiquan boxing[Title/Abstract] OR taiji exercise[Title/Abstract] OR taiji boxing[Title/Abstract] OR athletic taijiquan[Title/Abstract] OR taichi boxing[Title/Abstract] OR eight jin[Title/Abstract] OR eight brocades[Title/Abstract] OR eight brocade[Title/Abstract] OR eight sections jin[Title/Abstract] OR eight jin[Title/Abstract] OR Yi Jinjing[Title/Abstract] OR guiding technique[Title/Abstract] OR guidance method[Title/Abstract] OR guidance[Title/Abstract] |
| #5 | #3 AND #4 |
